# Learning ballet technique modulates the stretch reflex in students with cerebral palsy: case series

**DOI:** 10.1186/s12868-024-00873-0

**Published:** 2024-11-06

**Authors:** Citlali López-Ortiz, Maxine He, Deborah Gaebler-Spira, Mindy F. Levin

**Affiliations:** 1 Neuroscience Program, University of Illinois at Urbana-Champaign, 2325/21 Beckman Institute, 405 North Mathews Avenue, Urbana, IL 61801 USA; 2 Department of Kinesiology, University of Illinois at Urbana-Champaign, 906 South Goodwin Avenue, Urbana, IL 61801 USA; 3 Pediatric Rehabilitation, Shirley Ryan AbilityLab, 355 East Erie Street, Chicago, IL 60611 USA; 4 Department of Physical Medicine and Rehabilitation, Feinberg School of Medicine, Northwestern University, 710 North Lake Shore Drive, Chicago, IL 60611 USA; 5 School of Physical and Occupational Therapy, Faculty of Medicine and Health Sciences, McGill University, 3654 Prom Sir-William-Osler Street, Montreal, QC H3G 1Y5 Canada; 6 Centre for Interdisciplinary Research in Rehabilitation, Montreal, Canada

**Keywords:** Cerebral palsy, Ballet, Stretch reflex, Motor learning, Rehabilitation, Dance

## Abstract

**Background:**

Cerebral palsy (CP) is considered the most prevalent developmental motor disorder in children. There is a need for training programs that enhance motor abilities and normalize function from an early age. Previous studies report improved motor outcomes in dance interventions for CP. Investigating the neurophysiological mechanisms underlying such improvements is necessary for efficient and safe intervention design. This study reports changes in stretch reflex responses as the primary neurophysiological motor outcome of a targeted ballet class intervention.

**Results:**

A case series of participants with mixed spastic and dyskinetic CP (n = 4, mean age = 12.5 years, SD = 6.9S years, three female, one male) who learned ballet technique in a course of one-hour classes twice per week for six weeks is presented. Changes in stretch reflex responses and in clinical motor tests as secondary outcomes were observed after the course and at one-month follow-up. Quantitative measures of elbow or ankle stretch reflex were obtained using electromyography and electrogoniometry. The joint angle of the stretch reflex onset varied across velocities of stretch, and its variability decreased after the intervention. Within-subject tests of the central tendency of stretch reflex angle coefficients of variation and frequency distribution demonstrated significant changes (p-values < 0.05). Secondary outcomes included the Quality of Upper Extremity Skills Test (QUEST), Pediatric Balance Scale (PBS), Modified Tardieu Scale (MTS), Dyskinesia Impairment Scale (DIS), and Selective Control Assessment of the Lower Extremity (SCALE). All the participants demonstrated improvements larger than the minimal clinical important difference (MCID) or the smallest detectable difference (SDD), as applicable.

**Conclusions:**

Evidence of changes in the stretch reflex responses in these four cases of mixed CP was observed. The observed variability in the stretch reflex responses may be due to the dyskinetic component of the mixed CP presentations. More studies with a larger sample size and longer duration of learning and practice of ballet technique are necessary to establish the extent of possible modulation and adaptation of the stretch reflex response as a neurophysiological basis for observed improvements in clinical measures.

*Trial registration*: This study was registered in the Clinical Trials Protocol Registration and Results System (NCT04237506, January 17, 2020).

**Supplementary Information:**

The online version contains supplementary material available at 10.1186/s12868-024-00873-0.

## Introduction

Cerebral palsy (CP) is considered the most prevalent developmental motor disorder in children [1]. CP is considered a non-progressive condition consisting of a group of permanent dysfunctions in movement and posture resulting from injuries in the developing brain during the prenatal and perinatal periods [2]. According to the Surveillance of Cerebral Palsy working group in Europe, muscle tone in CP is divided into hypertonic and hypotonic types, further classified into three main groups based on motor signs: (1) spastic, (2) dyskinetic (divided further into dystonia and choreoathetosis), and (3) ataxic [3]. Hypertonic and hypotonic manifestations can be present in the same child as in the mixed presentation of spasticity and dyskinesia [4]. The motor impairments result in decreased motor performance and participation in social and daily living activities that evolve into increased disability with age [5]. Hence, there is a need to develop training programs that yield positive motor outcomes and normalize activities and participation throughout the lifespan [6].

Various dance forms, including creative dance, dance-based in somatic therapies, ballroom dance, hip-hop, and classical ballet, have shown positive movement outcomes for persons with CP while increasing social participation. Previous studies have reported improved balance, gait, selective motor control, range of motion, rhythm production, and executive function [5–9]. However, the neurophysiological mechanisms that underlie such improvements have not been elucidated. Gaining a better understanding of the underlying neurophysiological mechanisms is key for the objective, beneficial, and efficient design of dance classes and programs for persons with CP while avoiding possible motor rehabilitation pitfalls.

Several studies on stretch reflex adaptation in classical ballet dancers and modern dancers have shown that ballet and modern dance training produce a long-term depression of the H-reflex [10–15]. The H-reflex has the same neuronal pathway as the stretch reflex, but it is electrically induced as opposed to a mechanically induced muscle stretch. It is estimated that 80% of individuals with CP present heightened stretch reflexes that underlie clinical spasticity [4]. Thus, adaptation through learning and practicing ballet techniques in CP may constitute an important neurophysiological mechanism for the improved motor outcomes reported in the previous research [5, 14, 16–21]. This study quantifies the changes in the stretch reflex after learning classical ballet technique in a course similar to that of previously published studies as one plausible neurophysiological mechanism for the previously reported improved motor outcomes [19–21].

In the case of individuals with mixed spastic and dyskinetic CP, the heightened stretch reflexes may be dependent on joint angle alone, or on joint angle and joint velocity [4] and restrict the range of motion at the affected joint and its mechanical chains. The Montreal Spasticity Measure (MSM) provides a quantitative means of measuring the tonic stretch reflex threshold response, taking into account both joint angle and velocity [22].

In this context, studies on the stretch reflex using the MSM have aimed to characterize spasticity or rigidity in patients with stroke, spastic CP, and Parkinson’s disease [23–27]. To our knowledge, the tonic stretch reflex response of the MSM has not been investigated in individuals with spastic and dyskinetic CP. In order to study the potential adaptation of the stretch reflex responses due to learning ballet technique, we delivered an individualized ballet course to participants with mixed spastic and dyskinetic CP with velocity dependent (dynamic) stretch reflex threshold (DSRT) measurements as the primary outcome before the course, after the course, and at one-month follow-up. The tested hypotheses for each participant were: (1) variable DSRT responses characterize mixed CP, and (2) the distribution of the number of DSRT responses with respect to angular velocity differs from before to after participation in the ballet technique course, and at one-month follow-up.

## Methods

This interventional study consisted of a case series of individual within-subject pre-, post-, and one-month follow-up design. For the primary outcome measure, three days of DSRT measurements were included in each testing period. Secondary outcome measures were tested once per testing period. Secondary outcomes included clinical motor assessments of balance, range of motion, spasticity, and dyskinesia before, after, and at one-month follow-up. This study is reported following the STROBE guidelines [28].

### Participants

The study was approved by two local Institutional Review Boards and was registered in the Clinical Trials Protocol Registration and Results System (NCT04237506). All procedures performed in studies involving human participants were in accordance with the ethical standards of the institutional and/or national research committee and with the 1964 Helsinki Declaration and its later amendments or comparable ethical standards. The study was approved by the IRB of the University of Illinois at Urbana-Champaign (No. 16718) and Carle Foundation Hospital IRB (No. [983792-3] 16CNI1335) in a university town in a rural setting. The recruitment period started on November 5, 2016, and ended on April 30, 2020. Written informed consent from the parent and assent from the child, as well as consent from adult participants, was obtained before enrollment. The inclusion criteria were (1) ages 3 to 64, (2) have Gross Motor Function Classification System (GMFCS) level I to IV [29], (3) have no other neuromuscular or musculoskeletal conditions, (4) participate in stable school and/or private physical therapy with a regular frequency no greater than one session per week, (5) have the ability to pay attention and follow three-step directions, (6) be medically stable. The exclusion criteria were (1) have other neuromuscular or musculoskeletal conditions, (2) have had surgical procedures within six months prior to enrollment in the study, (3) have had changes in medication within six months prior to enrollment in the study, (4) have other concurrent illness, (5) have had botulinum toxin treatment within three months prior to enrollment in the study, and (6) have uncorrected vision. The clinical criteria were verified via a questionnaire completed by the participant’s physician. Other demographic information was self-reported by the participant or the participant’s parent. Participants were recruited by posting flyers, sending invitation letters to patients meeting the inclusion and exclusion criteria in the Level I Trauma and Level III local hospitals, and advertising in the local university's faculty and staff electronic newsletter.

### Sample size

The a priori sample size calculation was based on a statistical t-test on the difference between the means of matched pairs design, assuming an effect size dz = 0.8, the probability of a type I error = 0.05, and power = 0.8. Using matched pairs tests in which each participant serves as their own control allows for control of extraneous confounders that may be present and unidentified. This calculation yielded a total sample size of n = 12, and we proposed a sample size of n = 16 for IRB approval to account for possible attrition. Thus, the original enrollment goal was 16 participants.

### Intervention

The personalized ballet course intervention was delivered for a duration of one hour twice per week for six consecutive weeks. Each ballet class followed the guidelines developed by López-Ortiz et al. [19]. The structure of the classes was adapted from the Pre-Primary, Primary, and Level I Syllabi of the Royal Academy of Dance [30, 31]. In these levels, ballet technique focuses on training postural control and prioritizes lower limb training while maintaining position control in all joints. Anatomically safe positioning of all joints was monitored throughout the classes. Training of selective motor control (joint movement isolation and joint movement sequencing), external hip rotation, breath control, and core strength were targeted. The classes took place in a fully equipped dance studio with ballet barres, mirrors, a professional dance floor, a piano, and a sound system with pitch control. Live piano accompaniment or recorded ballet class music was used. Dance classes were delivered to each participant individually by an experienced certified ballet teacher with expertise in motor control research (CLO) and assisted by two to four trained assistants as needed to ensure fidelity in the delivery. The assistants were trained to support the specific needs of the participants while learning ballet technique safely. The assistants facilitated learning new coordination patterns through demonstration, physical guidance, physical support, and touch. This ensured that the participants were exposed to similar ballet technique content in a safe manner despite the differences in physical characteristics and ability. Dance improvisation was incorporated at the end of each class to enhance performance, expressive communication, and motivation. Theatrical props and costumes were available to the participants to use at will during dance improvisations. The one-hour targeted ballet classes were delivered twice weekly for six weeks (Week 1 to Week 6). If a class was missed, a make-up class was delivered within one week of the missed class.

### Quantitative measure of the dynamic stretch reflex response (DSRT)

Each participant was instructed to lay supine with the head resting on a pillow, the knees supported at approximately 15° of flexion, and to relax. After manually identifying the elbow or ankle with the most apparent spasticity, that joint was selected for DSRT testing. For elbow extension or ankle dorsiflexion, EMG electrodes (Thought Technology Ltd., Canada) were attached to the skin over motor points on the biceps long head and lateral triceps, or lateral gastrocnemius and tibialis anterior according to the motor point locations described by Leis and Schenk [32]. An electrogoniometer with two arms (servo-type rotational-position P2200; Novotechnik U.S. Inc., Southborough, US) was positioned close to the center of rotation of the joint tested, and the two arms were attached to the lateral aspects of the arm and forearm (for the elbow) or the shank and foot (for the ankle joint). All data were collected at a sampling rate of 1000 Hz. Full elbow extension and full ankle dorsiflexion were defined as 0°. Each participant was instructed to relax, and baseline EMG was acquired. Next, the participant was instructed to perform a maximal voluntary contraction with the joint positioned at 90°, and EMG was acquired for calibration. The participant was subsequently instructed to relax and return the EMG to the baseline level while the joint was positioned and maintained at the starting angle for the manual stretch response. This initial angle was recorded by the system and was matched (± 10°) prior to each stretch. The MSM system prompted the examiner to manually stretch the selected joint with evenly distributed slow, moderate, or fast velocity stretches guided by a series of tones produced by the software in random order. This procedure was repeated until the system detected 20 valid DSRT responses. The DSRT was valid when the EMG response to stretch exceeded three standard deviations from the mean baseline muscle activity for a minimum of 25 ms. The Montreal Spasticity Measure (MSM) provides a quantitative means of measuring the DSRT and then to determine the tonic stretch reflex threshold (TSRT) response [22], which is an abstract measure shown to have good reliability in patients with moderate to severe spasticity [26]. The tonic stretch reflex threshold (TSRT) is the extrapolated joint angle in which the activation of motor neurons would occur at rest (i.e., if the velocity of stretch was equal to zero) [33]. To determine the TSRT, stretch reflex onsets in response to a range of stretch velocities are evoked. During an imposed muscle stretch, the joint angle at which electromyography (EMG) activity increases is referred to as the dynamic stretch reflex threshold (DSRT) for each velocity of stretch. The TSRT is the intercept at the x-axis of the extrapolated linear regression line in the velocity of stretch vs. the DSRT measured values [23, 24]. This procedure was performed in three pre-assessment sessions during each testing week: Week 0 for baseline assessments, Week 7 for post-assessments, and Week 10 for one-month follow-up assessments. For further details on MSM procedures, please refer to Blanchette et al. and Mullick et al. [24, 25].

### Clinical measures

Clinical measures included the Quality of Upper Extremity Skills Test (QUEST) [34], Pediatric Balance Scale (PBS) [35], Modified Tardieu Scale (MTS) [36], Dyskinesia Impairment Scale (DIS) [37], Selective Control Assessment of Lower Extremity (SCALE) [38], Gross Motor Function Classification System (GMFCS) [39] and Manual Ability Classification System (MACS) [40]. These assessments were administered once in Weeks 0, 7, and 10 to determine clinical motor outcomes. Blinding is not possible for these clinical assessments; therefore, to avoid assessment bias, all procedures were videotaped, and the initial assessment results were verified independently by at least two trained raters until a rating consensus was reached.

The changes in clinical measures from Week 0 to 7 and from Week 0 to 10 were compared to the minimal clinical important difference (MCID) or smallest detectable difference (SDD) as appropriate to determine if the change obtained was clinically meaningful. For QUEST and PBS, higher values indicate better motor function, with MCIDs of 4.89 points and 5.83 points, respectively [34, 35]. For the MTS and DIS, the SDD was used [36, 37]. In the MTS, the SDD for angle of catch and range of motion at the elbow joints are 13.98° and 12.39°, respectively [36]. Since in the MTS full elbow extension is defined as 180◦, a larger value of angle of catch indicates less spasticity severity. DIS has two subscales, dystonia and choreoathetosis; the SDD for each is 15% and 7%, respectively [37]. A lower score indicates less severity of dystonia and/or choreoathetosis. Higher values in SCALE indicate better selective motor control [38], and radar plots of the SCALE enclosing a larger area by the radar plot line indicate less impairment.

### Statistical analysis

#### Quantitative measures of the stretch reflex responses

The statistical analyses for this case series focus on descriptive statistics and statistical hypothesis testing of each participant’s data individually. All individual within-subject statistical analyses consider each participant as their own statistical control, thereby eliminating possible individual confounding factors. All calculations were performed using Prism 8.3 (GraphPad, San Diego, CA). To address hypothesis 1: variable DSRT responses characterize mixed CP, a linear regression analysis of angular velocity vs. DSRT was performed for each testing session and participant, as reported in previous literature [41, 42]. Also, the coefficients of variation of the DSRT angular velocity responses were calculated at each bin of angular velocities with more than three values in Weeks 0, 7, and 10 for each participant. Box plots and descriptive statistics tables are presented for each participant in Fig. 2 (left column) and Supplementary Table S2, respectively. Shapiro–Wilk normality and equal variance tests were conducted for each participant’s week data. One-tailed within-subject student’s t-tests were performed on the coefficient of variation for each participant individually to compare mean variability from baseline to post-participation values. The Cohen’s d effect size was calculated when significant differences were present. To address hypothesis 2: the distribution of the number of DSRT responses with respect to angular velocity differs from before to after participation in the ballet technique course, and at one-month follow-up, one-tailed within-subject student’s t-tests were performed on individual participants’ data sets that passed the normality and equal variance tests. These tests compared the frequency distribution means of the DSRT responses with respect to angular velocity (bin = 10°/s) from baseline to post-participation values. One-tailed Welch’s t-tests were used for the data sets that passed the normality tests and had unequal variance. When significant differences were present, the Cohen’s d effect size was calculated for all t-tests. The data sets that did not pass normality tests were analyzed using a two-sample Kolmogorov–Smirnov test. A p-value < 0.05 was considered statistically significant.

## Results

### Participant enrollment

Study advertisement yielded six parties reaching out for enrollment. Six individuals were assessed for inclusion and exclusion criteria, and all were enrolled in the study. Two participants did not complete the study, one due to scheduling conflicts before post-testing could be initiated, and the other could not focus attention and follow instructions during Week 0 testing. We report the data on the four participants who completed at least the Week 7 assessment sessions. Of these, one did not participate in Week 10 assessments. The self-reported or parent-reported participant characteristics of the four individuals who completed the ballet technique course are presented in Table 1. In summary, four participants with mixed spastic and dyskinetic CP included in this case series, participants had mean age = 12.5 years, SD = 6.9 years, three were female, and one was male. The achieved enrollment numbers allow for a case series report using individual within-subject statistical analyses of the quantitative outcome measures.

**Table 1:** Participant characteristics

Personal Information				
Participant	1	2	3	4
Age	22	7	8	13
Gender	Female	Male	Female	Female
Origins of CP	Congenital	Prenatal trimester 2: spontaneous blood flow in the parietal and frontal lobe, microcephaly and poly microgyria	Not reported	Prenatal trimester 2
GMFCS	4	1	2	1
MACS	4	3	1	Left: 1, Right 3
Extremity Distribution	Quadriplegia and right-side neglect	Left hemiplegia	Mild Quadriplegia	Right hemiplegia and mild left hemiplegia
Medication	Not reported	Not reported	Note reported	Not reported
Frequency of Treatment	PT: once a week for 50 min, AT: twice per week for 15 min	PT: twice per week for 30 min	OT: 90 min per week, PT: 75 min per week, AT: 30 min per week, Writing at YMCA: 45 min per week	PT: once every other week for 50–60 min
Botulinum Toxin History	None	Once on arms/thumb	Multiple injections from 2011 to 2015	Multiple injections from 2007 to 2012, paired with serial casting
Surgical History	Twice on lower limbs	None	Tethered cord release	Right wrist tendon release
Other Health Condition	None	Seizures, attention deficit disorder	None	None
Type of CP	Spastic unilateral CP, dystonic CP	Spastic unilateral CP, dystonic CP, choreo-athetotic CP	Spastic unilateral CP, dystonic CP, choreo-athetotic CP	Spastic unilateral CP, dystonic CP, choreo-athetotic CP
Hypertonia	Spasticity, dystonia	Spasticity, dystonia	Spasticity, dystonia	Spasticity, dystonia
Hypotonia	Trunk hypotonia		Trunk hypotonia	
Hyperkinesia		Myoclonus (not spontaneous)	Myoclonus	Myoclonus
Participant’s Ability and Lifestyle				
Physical Activity Level Compared to Same-age Peers				
Academic Performance				
Reading Skills Compared to Same-age Peers	Above average	Below average	Above average	Above average
Mathematic Skills Compared to Same-age Peers	Average (keeps up)	Below average	Below average	Average (keeps up)
Writing Skills Compared to Same-age Peers	Above average	Cannot write/unable to write a sentence with correct spelling	Below average	Above average

All participants lived in Central Illinois. Once participant was lost due to scheduling conflicts and a second participant was excluded from the study in Week 0 due inability to focus attention and follow assessment instructions [5]. Demographic information from these two participants is excluded in Table 1. The classification of cerebral palsy follows the guideline of Surveillance of Cerebral Palsy in Europe [3]. *CP* cerebral palsy, *GMFCS* Gross Motor Function Classification System, *MACS* Manual Ability Classification System, *PT* physical therapy, *AT* aquatic therapy, *OT* occupational therapy

## Quantitative outcome measures

**Fig. 1:**
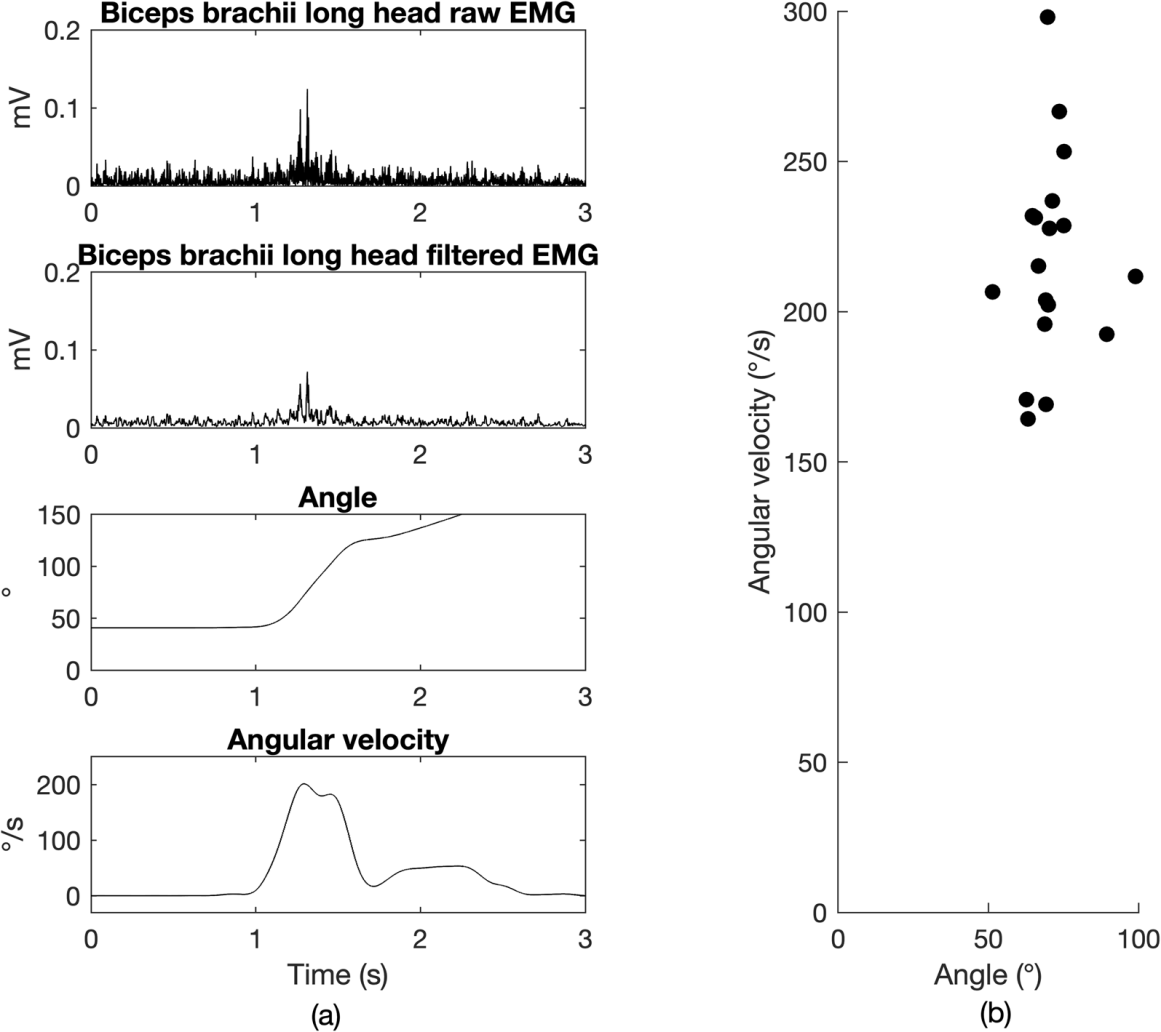
**a** Sample data from a single trial in session 5 of stretching elbow flexors from participant A. The raw and filtered EMG of biceps brachii long head are plotted in the top two panels. The third panel shows the change of angle as a function of time, and the bottom panel shows angular velocity vs. time. The DSRT angle is 65.75, and its corresponding angular velocity of stretch is 196.83/s. **b** Representative angular velocity vs. angle graph showing DSRTs for participant A in session 5. The plot shows that 20 DSRTs were evoked at various angular velocities in a single testing session. Similar angles are recorded for a large range of angular velocities, indicating the lack of velocity dependence of the stretch reflex, which would occur in spasticity. No significant linear relationship was found (p-value = 0.7514)

Representative data of a single stretch of the elbow flexors, including EMG, angle, and angular velocity vs. time, are presented in Fig. 1a. A representative example of angular velocity vs. angle is shown in Fig. 1b. Not all stretches resulted in a stretch reflex response. The percentages of imposed stretches that elicited a DSRT response are included in the Supplementary Figure S1. Linear regression analysis of angular velocity of stretch vs. angle resulted in low correlation coefficients *r* (< 0.6452), and 69.7% of total trials had non-significant p-values for the slopes of the linear fits, indicating the high variability in the responses as expected for mixed CP in hypothesis 1. These results are reported in Supplementary Table S1.

**Fig. 2:**
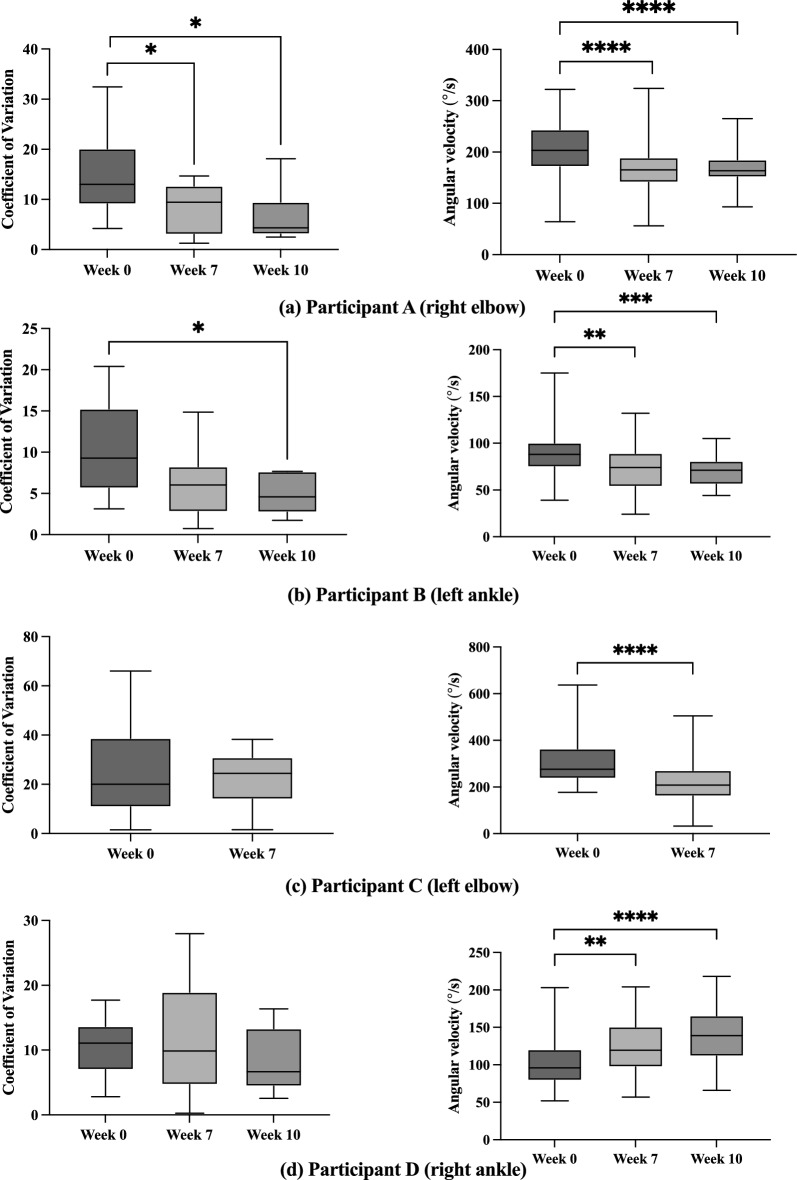
Box plots the coefficients of variation of the DSRT angles of catch (left column) and DSRT angular velocity responses (right column) for each participant. The box plots show the descriptive statistics values for the coefficient of variation of the DSRT angle and DSRT angular velocity for each participant across all trials on Weeks 0, 7, and 10, showing the minimum value, first quartile, median, third quartile, and maximum values measured. The normality and equal variance test results are presented in Supplementary Table S3. The corresponding frequency distribution plots are included in Supplementary Figures S2, S3, S4, and S5. The asterisks in the plots indicate significant differences in the corresponding statistical tests shown in Table 2. The asterisks in the graphs correspond to the following levels of significance: *p-value < 0.05, **p-value < 0.01, ***p-value < 0.001, ****p-value < 0.0001. For specific p-values, see Table 2

Exploring further the behavior in the variability of the DSRT responses, all measured values of the DSRT angles for each individual participant in Weeks 0, 7, and 10 are reported in detail in Supplementary Figures S2, S3, S4, and S5. The corresponding box plots of the coefficients of variation of the DSRT angles for each testing week are plotted in Fig. 2 (left column), and the descriptive statistics values are included in the Supplementary Table S2. The results of Shapiro Wilk normality tests and equal variance tests are included in the Supplementary Table S3. The p-values of one-tailed student’s t-tests of each participant on the coefficients of variation of the DSRT angles within each bin of velocity of stretch in Weeks 0, 7, and 10 were calculated and are reported in Table 2. The significant results are indicated by asterisks in Fig. 2 (left column). In summary, reduced means were obtained after the intervention and at one-month follow-up (Weeks 7 and 10) for participant A (W0 vs. W7: p-value = 0.0112, CI = 1.030 to 12.34, df = 25; effect size = 0.947; W0 vs. W10: p-value = 0.0127, CI = 1.118 to 15.27, df = 20, effect size = 1.142) and for participant B (W0 vs. W10, p-value = 0.0469, CI = -1.024 to 11.26, df = 11, effect size = 1.137); no significant reductions in the mean of the coefficients of variation were obtained for participants C and D.

**Table 2:** One-tailed student’s t-tests and Welch’s t-test were performed on the DSRT angular velocity data for participants A and B

One-tailed Student’s t-tests on DSRT coefficient of variation				
Participant	W0 vs. W7 p-values	Effect size Cohen’s d	W0 vs. W10 p-values	Effect size Cohen’s d
A	0.0112* (n_0_ = 14, n_7_ = 13)	0.947	0.0127* (n_0_ = 14, n_10_ = 8)	1.142
B	0.0558 (n_0_ = 8, n_7_ = 10)	–	0.0469* (n_0_ = 8, n_10_ = 5)	1.137
C	0.3524 (n_0_ = 16, n_7_ = 13)	–	N/A	–
D	0.2961 (n_0_ = 10, n_7_ = 13)	–	0.1272 (n_0_ = 10, n_10_ = 14)	–

One-tailed tests on DSRT angular velocity				
	One-tailed Student’s t-tests on DSRT angular velocity W0 vs. W7		One-tailed Welch’s t-test on DSRT angular velocity W0 vs. W10	
Participant	p-value	Effect size Cohen’s d	p-value	Effect size Cohen’s d
A	< 0.0001* (n_0_ = 60, n_7_ = 58)	0.796	< 0.0001* (n_0_ = 60, n_10_ = 40)	0.759
B	0.0005* (n_0_ = 30, n_7_ = 60)	0.616	0.0010* (n_0_ = 30, n_10_ = 40)	0.887
	Kolmogorov–Smirnov test on DSRT angular velocity			
	W0 vs. W7		W0 vs. W10	
C	< 0.0001* (n_0_ = 60, n_7_ = 55)		N/A	
D	0.0090* (n_0_ = 60, n_7_ = 60)		< 0.0001* (n_0_ = 60, n_7_ = 60)	

The asterisk indicates statistical significance (p-value < 0.05.)

The Kolmogorov-Smirnoff test was performed on the DSRT angular velocity data of participants C and D as a consequence of the results of the Shapiro–Wilk normality tests. The p-values are presented with the sample size for the corresponding week comparisons. The asterisk indicates statistical significance (p-value < 0.05.)

Addressing hypothesis 2, the frequency distribution plots for the DSRT angle corresponding to each bin of angular velocity of width = 10°/s for each participant were generated and are included in the Supplementary Figures S2, S3, S4, and S5. The corresponding box plots are presented in Fig. 2 (right column) and the descriptive statistics values are included in the Supplementary Table S2. The results of Shapiro–Wilk normality tests and equal variance tests on the data on each participant are included in the Supplementary Table S3. In accordance with the results of the normality and equal variance tests presented in the in the Supplementary Table S3, we report the results of one-tailed student t-tests, one-tailed Welch tests, or two-sample Kolmogorov–Smirnov tests as appropriate. Significant results of the student t-tests, Welch test, and two-sample Kolmogorov–Smirnov test for differences between the distributions are shown with asterisks in Fig. 2 (right column). Details of the statistical tests are included in Table 2. Briefly, reduced angular velocity means were obtained after the intervention and at one-month follow-up (Weeks 7 and 10) for participant A (W0 vs. W7: p-value < 0.0001, CI = 22.16°/s to 59.68°/s, df = 116; effect size = 0.796; W0 vs. W10: p-value < 0.0001, CI = 17.34°/s to 53.43°/s, df = 98, effect size = 0.759); for participant B (W0 vs. W7: p-value < 0.0005, CI = 4.706°/s to 27.96°/s, df = 88; effect size = 0.616; W0 vs. W10, p-value = 0.0010, CI = 8.493°/s to 30.96°/s, df = 68, effect size = 0.887). For participant C, the Kolmogorov-Smirnoff test showed significant differences in the distributions from Week 0 to Week 7, with a smaller median angular velocity on Week 7. For participant D, the Week 0 distribution did not pass the Shapiro–Wilk normality tests. Therefore, the two-sample Kolmogorov-Smirnoff test was used to compare the distributions from Week 0 to Week 7 and Week 0 to Week 10. The two-sample Kolmogorov-Smirnoff test showed significant differences in the distributions from Week 0 to Week 7 (p-value = 0.0090; n_0_ = 60, n_7_ = 60) and Week 0 to Week 10 (p-value < 0.001; n_0_ = 60, n_10_ = 60), with progressively increasing median DSRT angular velocities from Week 0 to Weeks 7 and 10 [43].

In summary, all the distributions changed significantly. Participant D demonstrated an increasingly larger DRST angle of catch after participation, and the rest of the participants showed smaller angles of catch.

### Clinical measures

The QUEST, PBS, MTS, and DIS results are presented in Supplementary Table S4. The changes in QUEST showed that participants A and B improved beyond the MCID, while the other two participants remained stable throughout. Only participant A gained an MCID in the PBS, while the scores for the rest of the participants were stable (see Supplementary Table S4). Participants A, B, and D showed a larger range of motion in one joint before the angle of catch of the MTS, while participant C showed improvement bilaterally at the elbow joints. Participant D demonstrated an increased range of motion only at the elbow of the least affected side. Improvement in DIS larger than the SDD was observed in three participants, with ameliorated dystonic scores in participants A, B, and D reflecting dystonic movements of shorter duration and smaller amplitude during activity. Participant A also showed ameliorated dystonic symptoms during rest beyond the SDD. For the choreoathetosis subscale, an SDD amelioration was observed in participants A and B. Radar plots of the sub-scores of SCALE for Weeks 0, 7, and 10 are presented in the Supplementary Figure S6. These graphs demonstrate improvement in selective motor control in three participants (B, C, D). Two participants (B and D) demonstrated unilateral improvement of selective motor control, and one showed bilateral enhancement, with the most improvement observed at the hip and subtalar joint.

## Discussion

This case series of four participants with mixed CP shows that despite the variability in clinical characteristics and the concomitant variability of DSRT responses at baseline, learning and practicing ballet technique for one hour twice per week for six weeks may reduce the variability of the DSRT responses and may change the angle of catch to more desirable values in some participants. We presented the descriptive statistics of each participant’s results and conducted the appropriate statistical inference tests to establish the significance and effect size of the changes elicited by the intervention in each individual case. The known stability of classic stretch reflex responses was absent in this set of four participants with mixed CP. This seems to indicate that dyskinesia introduces the high variability observed for the DSRT measurements in this study [27]. This high variability was captured by the lack of linearity in the angular velocity vs. angle graphs and by the coefficients of variation at each assessment period. Previous studies have reported that muscle activation during passive stretch was velocity-independent in patients clinically classified as mostly dystonic, and they had higher resistance to externally imposed movement at a low velocity [44]. This previous finding is consistent with the weak linear relationship between the angular velocity and DSRT angle observed in this study since dystonia was present in all participants, albeit it was not the most salient impairment. However, the variability decreased in all participants with respect to baseline after learning and practicing classical ballet technique. One participant showed depression in the DSRT response. Depression of the stretch reflex response naturally allows for a greater range of motion, which was observed in the same participant in the clinical MTS. A larger range of motion mechanically facilitates the motion of close-by joints and may contribute to the other improved clinical outcomes observed in SCALE and DIS.

The neurophysiological mechanisms that may underlie the results in this case series could be related to changes in the muscle spindle firing rates that signal proprioceptive information from muscles, primarily associated with changes in muscle length and are thus correlated with joint angle and joint angle velocity [45]. The sensitivity of muscle spindles to stretch is regulated by α-γ co-activation and variability in the strength of connections between muscle spindles and spinal neurons [46]. Descending motor pathways may modulate the activity of spinal neurons and result in depression of the stretch reflex. Other neurophysiological factors, such as tuning of the Golgi tendon organ, type of task, repeated testing, attentional shifts, and learning, can also modulate the stretch reflex response [45–47]. Any combination of these mechanisms may underlie the changes in the shift of DSRT response distributions with respect to angular velocity as measured before and after the intervention. Thus, the stretch reflex response measured in this case series may represent the aggregate result of these factors in mixed spastic and dyskinetic CP.

The changes in clinical outcome measures in these four individuals also revealed benefits in aspects of motor function, including upper limb function, balance, range of motion, and selective motor control of the lower limb. The improvements in selective motor control of the lower limb are most likely a reflection of learning ballet technique, which emphasizes the sequential activation of one joint at a time. Increased selective motor control is expected to lead to greater functional mobility and coordination. Notably, participant A, who had the most improvement, also had the highest level of GMFCS among the participants. This, along with the results of similar studies in CP, highlights the potential value of learning ballet technique for individuals with CP with higher levels of movement impairment. Additionally, amelioration of clinical dystonia and choreoathetosis as measured with the DIS was observed after participation in the targeted dance class, revealing further benefits of learning ballet technique in this cohort.

The individualized, targeted ballet technique class involved twelve hours of learning over six weeks. While this period was long enough to detect changes in quantitative measures and clinical outcomes, an intervention of longer duration may reveal larger and less variable results due to consolidation of learning and adaptation through practice. Dosage and long-term effects must be further investigated in more homogeneous and larger samples. Homogeneity of the sample in the CP population is extremely difficult to achieve. The natural variation in function and impairment contributions across individuals creates difficulty in cohort research in CP.

Although this study consists of a case series of four individuals, and it is impossible to generalize to other individuals with similar diagnoses, the descriptive statistics and inference tests highlight the variability and intricacy of changes in the stretch reflex for participants with mixed spastic and dyskinetic CP. The results of this case series also bring attention to a more fundamental need for the design of high-fidelity quantitative methods for characterizing movement in persons with CP, as most individuals have combined presentations of spasticity and dyskinesia, and there is high variability in the presentation of combinations. More sophisticated testing and quantitative analyses of these networks must be developed to fully capture the intricate responses of spinal motor neuron networks that regulate the stretch reflex response. Additionally, manual testing of the stretch reflex response involves the neuromechanical interaction between the tester and the participant, which can introduce extrinsic variables to the measurement. Blinded assessments of the clinical outcomes used are impossible; however, at least two independent assessors verified the values to avoid biases. Other factors, such as the participant's mood and fatigue, may also introduce variability in the measurements [4]. Therefore, more extensive research on motor learning and the practice of ballet technique interventions for the amelioration of clinical spastic and dyskinetic symptoms in CP is warranted.

In summary, more studies with larger sample sizes that could allow group comparisons in a randomized controlled trial, statistical methods that account for individual confounders, longer duration of learning and practicing ballet technique, and enhanced quantitative characterization of movement impairments are necessary to establish the extent of possible modulation and adaptation of the stretch reflex response as a neurophysiological basis for observed improvements in clinical outcomes. More research on this topic with large randomized controlled trials has the potential to empower the objective design of ballet technique classes, complementing traditional physical and occupational therapies and normalizing participatory activities in CP.

## Supplementary Information

Supplementary Material 1.

Supplementary Material 2.

Supplementary Material 3.

Supplementary Material 4.

Supplementary Material 5.

Supplementary Material 6.

Supplementary Material 7.

Supplementary Material 8.

Supplementary Material 9.

Supplementary Material 10.

## Data Availability

The de-identified data sets generated and/or analyzed during the current study are available from the corresponding author upon reasonable request.
