# Supplementary material for: Learning ballet technique modulates the stretch reflex in students with cerebral palsy: case series
Source: BMC Neurosci. 2024 Nov 6;25:66. doi: 10.1186/s12868-024-00873-0 (PMC11539840; doi:10.1186/s12868-024-00873-0)
Supplement: Supplementary file 1 — Supplementary Material 1. [file 12868_2024_873_MOESM1_ESM.pdf]

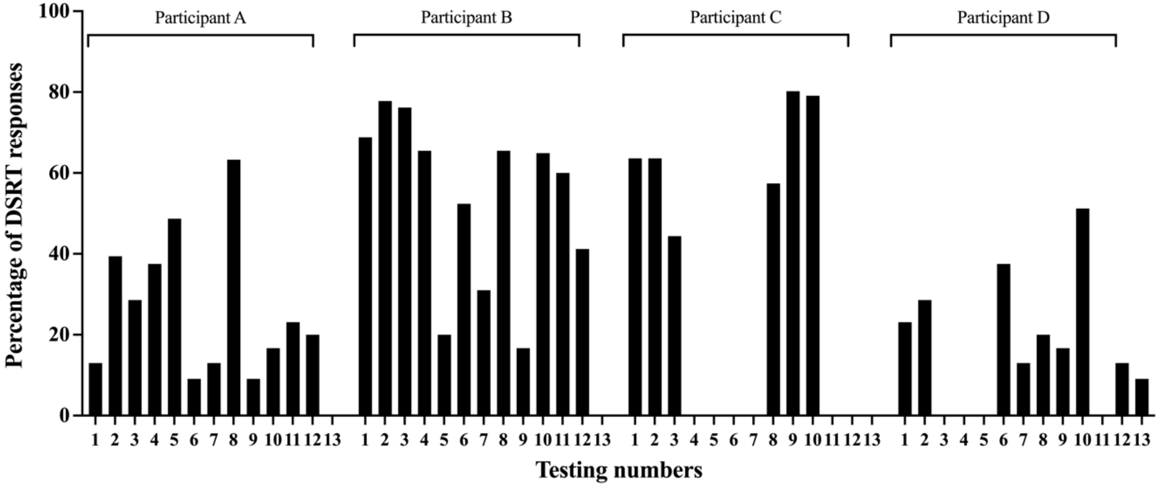

Figure S1. Percentage of DSRT responses. The bar charts show the percentages of successful trials in total numbers of trials in each testing session. Participant C completed three pre-assessments and three post-assessments only.
