# Supplementary material for: Learning ballet technique modulates the stretch reflex in students with cerebral palsy: case series
Source: BMC Neurosci. 2024 Nov 6;25:66. doi: 10.1186/s12868-024-00873-0 (PMC11539840; doi:10.1186/s12868-024-00873-0)
Supplement: Supplementary file 2 — Supplementary Material 2. [file 12868_2024_873_MOESM2_ESM.pdf]

Angular velocity (°/s)																							
55-65	86.9	51.6																					
65-75	84.5																						
75-85	45.7	46.7																					
85-95	62.8																						
95-105	48.5	49.4																					
105-115	76.2	NC																					
115-125	86.1	94.0	NC	45.0	47.8	48.1																	
125-135	60.4	69.3	NC	NC	49.1	49.4	60.7	NC	NC	52.4	54.5												
135-145	126.3	NC	NC	NC	NC	48.9	57.5	58.4	61.3	NC	NC	NC	NC	52.7	57.2	61.6							
145-155	87.9	NC	44.5	49.0	49.7	51.0	51.1	52.7	62.9	63.5	64.3	68.6	51.4	54.4	57.1	57.3	64.0	66.9					
155-165	63.2	66.0	78.8	NC	46.7	49.1	59.2	NC	51.4	57.1	58.2	59.5	65.1	68.3	86.8	NC							
165-175	62.6	64.3	69.2	70.5	71.4	91.1	NC	39.9	45.8	51.6	51.7	52.7	53.1	58.8	NC	NC	52.8	54.0	55.5	55.6			
175-185	69.9	74.2	NC	51.8	55.6	57.0	60.2	61.1	62.8	64.7	NC	NC	53.4	55.0	56.5	58.4	58.5	NC	NC				
185-195	62.2	64.8	66.6	67.4	72.5	89.4	NC	NC	49.9	53.4	NC	NC	NC	NC	54.8	55.2	58.0	59.2	NC	NC	NC	NC	NC
195-205	57.4	59.2	68.8	69.1	70.0	46.2	55.7	55.9	59.7	69.5	NC	NC	64.1	NC	NC								
205-215	51.4	70.8	99.0	NC	48.7	50.4	NC	NC	NC	NC	NC												
215-225	66.7	NC	NC	NC	56.1	60.0	61.6	NC															
225-235	59.5	60.5	64.7	65.6	66.9	70.4	75.1	NC	NC	53.2	62.3	NC	NC										
235-245	57.6	60.0	71.3	108.6	NC	80.2	NC	69.5															
245-255	65.4	71.1	75.2	102.5	NC	NC	58.8	NC	65.3														
255-265	59.0	NC	NC	NC																			
265-275	55.5	73.6	NC	49.9	NC	55.3																	
275-285	NC																						
285-295	53.6	57.8	60.3	67.7	NC																		
295-305	55.6	69.7																					
305-315																							
315-325	70.8	NC	61.7	NC																			
325-335	NC																						
335-345																							
345-355	NC																						

Figure S2. Distribution of DSRTs in participant A at the right elbow joint. The leftmost column represents intervals of angular velocity, with bin width of 10 degree/second. The actual angle values of each DSRT that was evoked at a specific velocity are reported in each colored cell. Data include repeated measures of the three testing sessions in Weeks 0, 7, and 10. DSRTs obtained in Week 0 are colored in light gray, DSRTs obtained in Week 7 are colored in medium gray, and DSRTs obtained in Week 10 are colored in dark gray. The interval of each bin includes the left endpoint while the right endpoint is excluded.
