# Supplementary material for: Learning ballet technique modulates the stretch reflex in students with cerebral palsy: case series
Source: BMC Neurosci. 2024 Nov 6;25:66. doi: 10.1186/s12868-024-00873-0 (PMC11539840; doi:10.1186/s12868-024-00873-0)
Supplement: Supplementary file 3 — Supplementary Material 3. [file 12868_2024_873_MOESM3_ESM.pdf]

Angular velocity (°/s)																						
Week 0																						
0-15																						
15-25																						
25-35																						
35-45	139.8																					
45-55	108.4	122.8																				
55-65	132.8	143.1	NC	NC	NC																	
65-75	103.6	138.6	NC	NC	NC	NC	NC	NC	NC													
75-85	113.4	124.5	138.0	143.6	NC	NC	NC	NC	NC	NC	NC	NC	NC	NC	NC	NC						
85-95	127.8	135.5	136.9	137.6	139.1	139.2	NC	NC	NC	NC	NC	NC										
95-105	106.3	117.7	121.2	123.9	134.5	137.4	141.1	NC	NC	NC	NC	NC	NC	NC	NC	NC						
105-115	110.3	140.2	NC	NC	NC	NC	NC	NC	NC	NC	NC	NC	NC	NC	NC	NC						
115-125	NC	NC	NC	NC	NC	NC	NC	NC	NC	NC	NC	NC	NC									
125-135	129.9	141.0	148.9	NC	NC	NC	NC	NC	NC	NC	NC	NC										
135-145	NC	NC	NC	NC	NC	NC																
145-155	NC	NC	NC	NC	NC																	
155-165	NC	NC	NC																			
165-175	121.2	NC																				
175-185																						
185-195	NC																					
195-205																						
205-215																						
215-225																						
225-235																						
235-245																						
245-255																						
Week 7																						
0-15	NC																					
15-25	140.6																					
25-35	141.8																					
35-45	139.9	140.1	140.7	142.2																		
45-55	111.2	132.6	135.2	135.6	139.4	139.9	140.2	141.2	147.0	NC	NC	NC	NC	NC								
55-65	131.7	132.9	135.4	135.6	137.3	139.6	140.4	141.6	141.8	NC	NC	NC	NC	NC	NC	NC	NC	NC	NC	NC		
65-75	129.7	131.9	136.3	136.6	136.9	139.2	139.3	141.0	NC	NC	NC	NC	NC	NC	NC	NC	NC					
75-85	122.9	131.9	134.2	134.8	136.8	137.3	138.0	138.6	141.6	144.7	144.9	149.6	NC	NC	NC	NC	NC	NC	NC			
85-95	106.7	129.3	131.4	134.4	143.9	NC	NC	NC	NC	NC	NC	NC	NC	NC	NC	NC	NC	NC	NC			
95-105	111.5	137.6	NC	NC	NC	NC	NC	NC	NC													
105-115	121.8	136.2	139.9	NC	NC	NC	NC	NC														
115-125	125.1	129.9	129.9	136.8	NC	NC																
125-135	125.9	139.0	NC	NC																		
135-145	NC	NC																				
145-155	NC	NC	NC	NC	NC																	
155-165																						
165-175																						
175-185																						
185-195																						
195-205																						
205-215																						
215-225																						
225-235																						
235-245																						
245-255	NC																					
Week 10																						
0-15																						
15-25																						
25-35																						
35-45	149.4																					
45-55	145.5	146.9	147.2	147.4	147.4	148.4	152.0	152.9	NC	NC	NC	NC	NC	NC	NC	NC						
55-65	131.4	141.9	141.9	143.8	143.8	144.2	148.6	NC	NC	NC	NC	NC	NC	NC								
65-75	131.8	133.3	135.6	136.8	139.1	140.7	146.0	146.2	148.8	149.3	NC	NC	NC	NC	NC	NC	NC	NC	NC	NC	NC	NC
75-85	131.2	133.2	136.7	140.4	141.0	161.0	NC	NC	NC	NC	NC	NC	NC	NC	NC	NC	NC					
85-95	125.9	131.0	134.2	137.6	139.2	156.6	NC	NC														
95-105	149.4	NC	NC																			
105-115	155.2																					
115-125																						
125-135																						
135-145																						
145-155	NC																					
155-165																						
165-175	NC																					
175-185	NC																					
185-195																						
195-205																						
205-215																						
215-225																						
225-235																						
235-245																						
245-255																						

Figure S3. Distribution of DSRTs in participant B at the left ankle joint. The leftmost column represents intervals of angular velocity, with bin width of 10 degree/second. The actual angle value of DSRT that was evoked at a specific velocity reported in each colored cell. DSRTs obtained in Week 0 are colored in light gray, DSRTs obtained in Week 7 are colored in medium gray, and DSRTs obtained in Week 10 are colored in dark gray. The interval of each bin is inclusive to the left endpoint is included while the right endpoint is excluded.
