# Supplementary material for: Learning ballet technique modulates the stretch reflex in students with cerebral palsy: case series
Source: BMC Neurosci. 2024 Nov 6;25:66. doi: 10.1186/s12868-024-00873-0 (PMC11539840; doi:10.1186/s12868-024-00873-0)
Supplement: Supplementary file 5 — Supplementary Material 5. [file 12868_2024_873_MOESM5_ESM.pdf]

Angular velocity (°/s)																							
05-15	NC																						
15-25																							
25-35																							
35-45	NC																						
45-55	90.1	NC	NC	NC																			
55-65	88.6	98.8	99.9	117.7	69.2	103.3																	
65-75	85.9	97.3	106.3	114.0	118.9	119.8	NC	110.9	NC	85.6	92.5	110.8											
75-85	90.5	95.3	95.5	96.7	100.7	103.6	107.8	110.3	114.7	121.3	121.7	84.5	102.0	124.1	NC	99.4	112.0						
85-95	93.0	114.1	114.2	114.7	116.0	118.3	NC	79.2	82.4	102.5	112.4	116.0	116.7	122.4	NC	NC	NC	83.4	85.5	110.5			
95-105	87.9	101.1	112.7	113.2	114.5	114.9	116.3	NC	NC	72.0	86.5	94.9	112.0	119.5	120.1	120.7	NC	NC	94.2	113.7			
105-115	77.7	95.9	101.9	118.3	121.1	68.7	78.4	92.4	117.4	118.3	118.3	121.9	NC	87.7	99.6	102.4	109.2	112.1	113.0	113.4	113.8	119.3	
115-125	88.8	89.9	93.1	96.4	99.6	114.6	116.8	116.9	119.6	NC	69.4	99.1	104.9	110.2	112.2	114.3	NC	NC	NC	NC	81.6	96.5	113.3
125-135	105.0	118.6	120.4	121.0	NC	98.4	105.1	115.0	115.3														
135-145	100.5	102.0	106.4	90.8	110.3	110.4	112.9	113.8	NC	NC	NC	NC	NC	110.5	113.2	113.9	114.6	115.6	120.2	122.8	123.5	124.8	126.0
145-155	96.7	124.4	NC	94.9	100.7	112.6	113.9	114.5	NC	114.8	120.0												
155-165	NC	91.6	96.9	111.1	114.6	115.6	115.9	NC	112.5	112.9	113.5	117.1	119.1	121.5	122.7								
165-175	110.7	111.1	112.3	113.1	113.6	125.4																	
175-185	110.3	113.4	116.7	111.9	112.0	113.1	NC	NC	110.0	121.2	122.1												
185-195	118.0	NC	94.6	108.6	113.3	124.2	125.9	127.9	NC	NC													
195-205	115.1	97.2	NC	NC	120.0	124.5																	
205-215	124.6																						
215-225	123.6	NC																					
225-235	NC																						
235-245	NC																						
245-255	NC	NC																					
255-265																							
265-275																							
275-285	NC	NC	NC																				

Figure S5. Distribution of DSRTs in participant D at the right ankle joint. The leftmost column represents intervals of angular velocity, with bin width of 10 degree/second. The actual angle value of DSRT that was evoked at a specific velocity reported in each colored cell. DSRTs obtained in Week 0 are colored in light gray, DSRTs obtained in Week 7 are colored in medium gray, and DSRTs obtained in Week 10 are colored in dark gray. The interval of each bin is inclusive to the left endpoint is included while the right endpoint is excluded.
