# Supplementary material for: Learning ballet technique modulates the stretch reflex in students with cerebral palsy: case series
Source: BMC Neurosci. 2024 Nov 6;25:66. doi: 10.1186/s12868-024-00873-0 (PMC11539840; doi:10.1186/s12868-024-00873-0)
Supplement: Supplementary file 6 — Supplementary Material 6. [file 12868_2024_873_MOESM6_ESM.pdf]

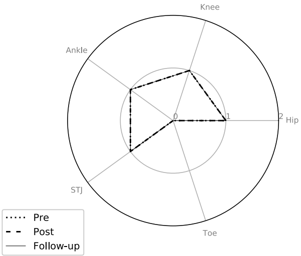

(a) Participant A (right)

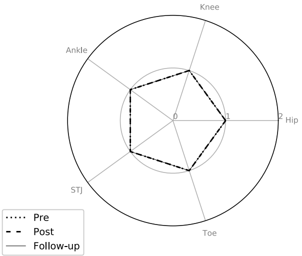

(b) Participant A (left)

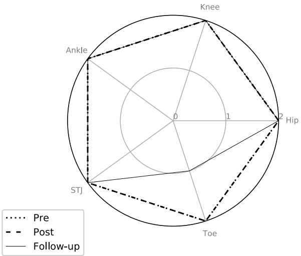

(c) Participant B (right)

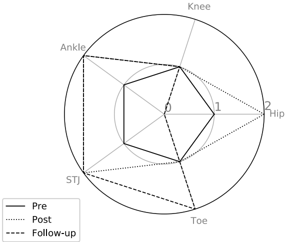

(d) Participant B (left)

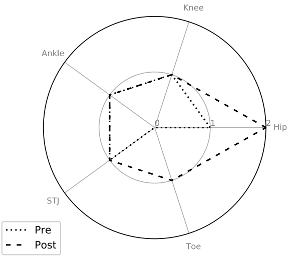

(e) Participant C (right)

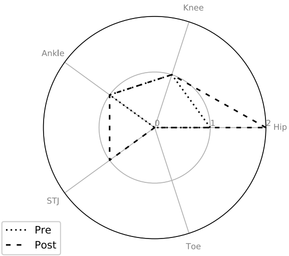

(f) Participant C (left)

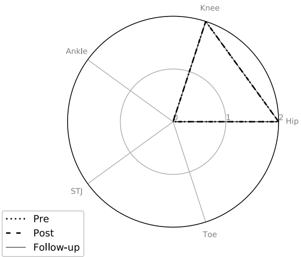

(g) Participant D (right)

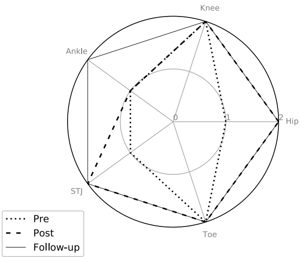

(h) Participant D (left)

Figure S6. Results of Selective Control Assessment of Lower Extremity (SCALE). The distribution of scores for each joint at three assessments were plotted for right and left lower limbs. Score 2 indicates normal selective control at the joint and score 0 indicates inability to perform isolated movement at that joint. Higher scores represent less impairment in selective motor control (34). The larger the area enclosed by the radar plot line, the less the impairment is. STJ: subtalar joint.
