# Supplementary material for: Learning ballet technique modulates the stretch reflex in students with cerebral palsy: case series
Source: BMC Neurosci. 2024 Nov 6;25:66. doi: 10.1186/s12868-024-00873-0 (PMC11539840; doi:10.1186/s12868-024-00873-0)
Supplement: Supplementary file 7 — Supplementary Material 7. [file 12868_2024_873_MOESM7_ESM.pdf]

Table S1. Linear regression of DSRT angular velocity vs. angle in each testing session.

	Participant A		Participant B		Participant C		Participant D	
Testing Session	r^2	p-value	r^2	p-value	r^2	p-value	r^2	p-value
1	0.2676	0.0195*	0.1464	0.2751	0.6789	< 0.0001*	0.1422	0.1012
2	0.0201	0.5510	0.0001	0.9764	0.0200	0.5512	0.3159	0.0099*
3	0.0096	0.3183	0.0005	0.9497	0.2468	0.0258*	0.0334	0.4406
4	0.0774	0.2350	0.0234	0.6728			0.1003	0.1737
5	0.0057	0.7514	0.1574	0.0833			0.0415	0.3893
6	0.0710	0.2560	0.4163	0.0021*			0.3094	0.0109*
7	0.2547	0.0232	0.3046	0.0116*			0.0509	0.3391
8	0.3270	0.0132*	0.0033	0.8110	0.4188	0.0020*	0.0403	0.3963
9	0.2481	0.0254	0.1422	0.1012	0.2051	0.0781	0.3184	0.0096*
10	0.0169	0.5853	0.4198	0.0020*	0.0285	0.4893	0.1897	0.0549
11	0.0089	0.6917	0.2806	0.0163*			0.1558	0.0850
12	0.0094	0.6839	0.1275	0.1222			0.0863	0.2087
13							0.2386	0.0289*

H0: regression slope = 0, statistical significance of p-value set at $p < 0.05$.
