# Supplementary material for: Learning ballet technique modulates the stretch reflex in students with cerebral palsy: case series
Source: BMC Neurosci. 2024 Nov 6;25:66. doi: 10.1186/s12868-024-00873-0 (PMC11539840; doi:10.1186/s12868-024-00873-0)
Supplement: Supplementary file 8 — Supplementary Material 8. [file 12868_2024_873_MOESM8_ESM.pdf]

Table S2. Descriptive statistics of DSRT coefficient of variation and DSRT angular velocity data.

Descriptive Statistics of DSRT Coefficient of Variation (a.u.)								
Participant	Week	Mean	Std. Dev	Min	Q1	Median	Q3	Max
A	0	14.92	8.67	4.21	9.08	13.01	20.13	32.44
	7	8.23	4.94	1.28	3.03	9.46	12.68	14.68
	10	6.72	5.28	2.47	3.10	4.36	9.47	18.11
B	0	10.19	5.82	3.13	5.64	9.29	15.27	20.41
	7	6.22	4.20	0.74	2.78	6.03	8.25	14.86
	10	5.07	2.58	1.74	2.73	4.60	7.66	7.67
C	0	24.32	17.72	1.51	10.86	20.02	38.72	66.05
	7	22.14	11.42	1.54	13.99	24.46	30.88	38.21
D	0	10.56	5.03	2.82	6.99	11.08	13.67	17.72
	7	12.24	8.68	0.26	4.69	9.88	18.93	27.96
	10	8.19	4.79	2.56	4.44	6.66	13.31	16.36
Descriptive statistics of DSRT angular velocity (°/s)								
Participant	Week	Mean	Std. Dev	Min	Q1	Median	Q3	Max
A	0	205.8	55.84	64.0	171.0	203.0	243.8	322.0
	7	164.9	46.43	56.0	140.8	165.0	189.0	324.0
	10	170.4	35.06	93.0	151.3	163.5	185.0	265.0
B	0	89.7	27.43	39.0	74.75	88.0	100.3	175.0
	7	73.37	25.52	24.0	53.75	74.0	89.25	132.0
	10	69.98	15.40	44.0	56.0	71.0	80.75	105.0
C	0	313.0	108.8	177.0	237.0	276.5	363.5	637
	7	215.9	89.04	32.0	161.0	208.0	271.0	505.0
D	0	103.6	34.36	52.0	79.25	96.0	120.3	203.0
	7	124.8	35.01	57.00	97.25	119.5	150.8	204.0
	10	138.1	37.22	66.00	111.5	139.0	165.5	218.0
