# Supplementary material for: Learning ballet technique modulates the stretch reflex in students with cerebral palsy: case series
Source: BMC Neurosci. 2024 Nov 6;25:66. doi: 10.1186/s12868-024-00873-0 (PMC11539840; doi:10.1186/s12868-024-00873-0)
Supplement: Supplementary file 9 — Supplementary Material 9. [file 12868_2024_873_MOESM9_ESM.pdf]

Table S3. P-values for the Shapiro-Wilk normality tests and equal variance tests on the DSRT coefficients of variation and angular velocity distributions.

DSRT coefficient of variation normality test p-values			
Participant	W0	W7	W10
A	0.0709	0.1065	0.0283*
B	0.4963	0.5313	0.4152
C	0.1704	0.6584	N/A
D	0.4184	0.4794	0.0739
DSRT angular velocity normality test p-values			
Participant	W0	W7	W10
A	0.6806	0.0606	0.1111
B	0.1090	0.0940	0.2007
C	<0.0001*	0.0283*	N/A
D	0.0010*	0.3695	0.6730

DSRT coefficient of variation equal variance test p-values		
Participant	Ho: Var(W0) = Var (W7)	Ho: Var(W0) = Var (W10)
A	0.0604	0.1928
B	0.3583	0.1343
C	0.1318	N/A
D	0.8500	0.1089
DSRT angular velocity equal variance test p-values		
Participant	Ho: Var(W0) = Var (W7)	Ho: Var(W0) = Var (W10)
A	0.1634	0.0026*
B	0.6270	0.0009*
	Ho: Var(W7) = Var (W10)	
D	0.6390	

The null hypotheses of normality of the DSRT angular velocity distributions were rejected at a p-value < 0.05 only for participant C in Weeks 0 and 7 and for participant D on Week 0. The null hypothesis of equal variance was rejected for p-value < 0.05 for the DSRT angular velocity of participants A and B for the comparison between Week 0 and Week 10.
