# Supplementary material for: Learning ballet technique modulates the stretch reflex in students with cerebral palsy: case series
Source: BMC Neurosci. 2024 Nov 6;25:66. doi: 10.1186/s12868-024-00873-0 (PMC11539840; doi:10.1186/s12868-024-00873-0)
Supplement: Supplementary file 10 — Supplementary Material 10. [file 12868_2024_873_MOESM10_ESM.pdf]

Table S2. Clinical assessment results of QUEST, PBS, MTS, and DIS.

Assessments	Subscores	Participant A			Participant B			Participant C			Participant D		
		W0	W7	W10	W0	W7	W10	W0	W7	W10	W0	W7	W10
QUEST	N/A	27	**34**†	**42**†	48	**57**†	**59**†	84	79	NT	84	86	85
PBS	N/A	33	**44**†	**39**†	51	52	53	47	47	NT	56	56	56
MTS Angle of catch	Right elbow flexor	NC	59	46	NC	NC	NC	30	**NC**†	NT	80	**51**†	70
	Right elbow extensor	NC	NC	60	88	**NC**†	**NC**†	NC	NC	NT	NC	NC	99
	Left elbow flexor	90	**NC**†	**52**†	NC	35	NC	65	**45**†	NT	NC	NC	NC
	Left elbow extensor	NC	NC	NC	42	**NC**†	**NC**†	NC	NC	NT	NC	NC	64
MTS Range of motion	Right elbow	140	150	139	144	140	156	130	140	NT	145	145	148
	Left elbow	150	148	134	154	154	162	140	120	NT	144	156	**157**†
DIS - Dystonia (action)	Duration	37	44	**28**†	31	**25**†	**24**†	16	NT	NT	27	**22**†	**21**†
	Amplitude	36	43	**26**†	31	**17**†	**24**†	10	NT	NT	20	**17**†	**14**†
DIS - Dystonia (rest)	Duration	24	**12**†	24	0	0	0	0	NT	NT	2	2	2
	Amplitude	13	**11**†	13	0	0	0	0	NT	NT	2	2	2
DIS - Choreoathetosis (action)	Duration	20	**13**†	22	4	**1**†	4	13	NT	NT	0	0	0
	Amplitude	17	18	21	4	**1**†	4	15	NT	NT	0	0	0
DIS - Choreoathetosis (rest)	Duration	0	0	4	0	0	0	0	NT	NT	0	1	0
	Amplitude	0	0	1	0	0	0	0	NT	NT	0	2	0

†The change in values, compared to Week 0, is equal to or larger than the minimal clinical important difference (MCID) or Smallest Detectable Difference (SDD) of each measure. MCIDs for QUEST and PBS are 4.89 points and 5.83 points, respectively (32, 33). SDDs for angle of catch and range of Motion in the MTS are 13.98º and 12.39º, respectively (34). SDDs for the DIS - Dystonia subscale is 15% and 7% for DIS - Choreoathetosis subscale (35). W0: Week 0; W7: Week 7; W10: Week 10; NT: not tested, NC: no catch detected.
