# Supplementary material for: Learning ballet technique modulates the stretch reflex in students with cerebral palsy: case series
Source: BMC Neurosci. 2024 Nov 6;25:66. doi: 10.1186/s12868-024-00873-0 (PMC11539840; doi:10.1186/s12868-024-00873-0)
Supplement: Supplementary file 4 — Supplementary Material 4. [file 12868_2024_873_MOESM4_ESM.pdf]

Angular velocity (°/s)																			
25-35	68.8																		
35-45	54.1	NC	NC	NC															
45-55																			
55-65																			
65-75	59.9	NC																	
75-85	NC																		
85-95	NC																		
95-105	101.0	NC																	
105-115	NC	NC	NC	NC															
115-125	NC	NC																	
125-135	62.4	108.6	NC	NC															
135-145	62.6	62.6	111.4	NC	NC	NC													
145-155	81.1	96.6	NC	NC	NC	NC													
155-165	NC	70.6	75.7	81.7	83.1	111.6	NC	NC	NC	NC	NC	NC							
165-175	NC	56.0	66.1	76.0	102.2	NC	NC	NC	NC	NC									
175-185	61.8	63.1	NC	72.7	NC	NC	NC	NC	NC	NC	NC								
185-195	50.6	88.3	57.7	72.1	NC	NC	NC	NC	NC										
195-205	73.9	NC	51.5	71.8	79.5	112.2	NC	NC	NC	NC	NC								
205-215	63.7	70.1	137.2	NC	64.0	78.2	89.7	107.9	112.8	127.6	NC	NC	NC	NC	NC	NC			
215-225	59.6	67.4	NC	107.1	146.8	NC	NC	NC	NC	NC	NC	NC	NC	NC					
225-235	61.3	69.2	69.8	81.0	NC	NC	NC	70.2	104.1	123.4	NC	NC	NC	NC	NC	NC	NC	NC	NC
235-245	65.6	125.8	NC	NC	NC	77.7	NC	NC	NC	NC	NC	NC	NC	NC					
245-255	65.5	71.1	NC	NC	114.5	117.0	NC	NC	NC	NC	NC								
255-265	55.4	63.9	64.2	66.1	68.0	78.2	80.4	NC	NC	NC	NC	NC	NC	NC					
265-275	56.3	65.2	69.6	NC	NC	NC	NC	107.2	164.1	NC	NC	NC	NC	NC	NC	NC	NC		
275-285	49.6	63.9	64.1	69.7	124.1	NC	71.2	NC	NC	NC	NC								
285-295	48.5	66.3	NC	85.9	NC	NC	NC	NC	NC	NC									
295-305	52.9	68.3	88.0	NC	70.1	73.7	NC	NC	NC	NC	NC	NC	NC						
305-315	72.8	NC	NC	83.0	NC	NC	NC	NC											
315-325	57.1	157.0	NC	NC	118.1	NC	NC	NC	NC	NC	NC	NC							
325-335	50.3	NC	90.3	NC	NC	NC													
335-345	52.6	NC	NC	160.3	NC	NC	NC	NC	NC										
345-355	NC	NC	75.6	NC	NC	NC	NC	NC											
355-365	61.7	67.9	97.3	NC	NC	NC	107.5	NC	NC	NC									
365-375	57.9	62.0	80.7	NC	NC	NC	NC	NC											
375-385	NC	NC																	
385-395	NC	NC																	
395-405	57.5	NC	NC	NC	NC	NC	NC	NC											
405-415	NC	NC	NC	NC	NC														
415-425	NC	NC	NC																
425-435	54.5	65.0	73.6																
435-445	59.4	NC	NC																
445-455	NC	NC	130.2	NC															
455-465	NC																		
465-475	NC	NC																	
475-485	NC																		
485-495	NC																		
495-505	59.7																		
505-515	NC	143.5																	
515-525	NC																		
525-535	65.8	NC																	
535-545	NC	NC																	
545-555	66.7	NC																	
555-565	NC																		
565-575	84.4	NC																	
575-585	NC	NC																	
585-595																			
595-605	NC																		
605-615	NC																		
615-625	NC																		
625-635	70.5																		
635-645	84.9																		
645-655																			
655-665	NC	NC																	
665-675	NC																		
675-685	NC																		
685-695																			
695-705																			
705-715	NC	NC	NC																
715-725																			
725-735	NC																		
735-745	NC	NC	NC																
745-755	NC																		
755-765	NC																		
765-775	NC																		
775-785																			
785-795	NC																		
795-805																			
805-815	NC																		
815-825	NC	NC																	
825-835																			
835-845																			
845-855																			
855-865	NC																		
865-875																			
875-885	NC																		
885-895																			
895-905																			
905-915	NC																		
915-925																			
925-935																			
935-945																			
945-955	NC																		

Figure S4. Distribution of DSRT for participant C at the left elbow joint. The leftmost column represents intervals of angular velocity, with bin width of 10 degree/second. The actual value of DSRT that was evoked at a specific velocity reported in each colored cell. DSRTs obtained in Week 0 are colored in light gray, and DSRTs obtained in Week 7 are colored in dark gray. The two rightmost columns represent the range of DSRT in Weeks 0 and 7 for each bin, and blank cells in these two columns indicate that no range can be calculated. The interval of each bin is inclusive to the left endpoint is included while the right endpoint is excluded.
